# Cardiomyocyte MEA Data Analysis (CardioMDA) – A Novel Field Potential Data Analysis Software for Pluripotent Stem Cell Derived Cardiomyocytes

**DOI:** 10.1371/journal.pone.0073637

**Published:** 2013-09-19

**Authors:** Paruthi Pradhapan, Jukka Kuusela, Jari Viik, Katriina Aalto-Setälä, Jari Hyttinen

**Affiliations:** 1 Department of Electronics and Communications Engineering, Tampere University of Technology, Tampere, Finland; 2 Institute of Biomedical Technology, University of Tampere, Tampere, Finland; 3 BioMediTech, Tampere, Finland; 4 HeartCenter, TampereUniversityHospital, Tampere, Finland; Indian Institute of Toxicology Research, India

## Abstract

Cardiac safety pharmacology requires in-vitro testing of all drug candidates before clinical trials in order to ensure they are screened for cardio-toxic effects which may result in severe arrhythmias. Micro-electrode arrays (MEA) serve as a complement to current in-vitro methods for drug safety testing. However, MEA recordings produce huge volumes of data and manual analysis forms a bottleneck for high-throughput screening. To overcome this issue, we have developed an offline, semi-automatic data analysis software, ‘Cardiomyocyte MEA Data Analysis (CardioMDA)’, equipped with correlation analysis and ensemble averaging techniques to improve the accuracy, reliability and throughput rate of analysing human pluripotent stem cell derived cardiomyocyte (CM) field potentials. With the program, true field potential and arrhythmogenic complexes can be distinguished from one another. The averaged field potential complexes, analysed using our software to determine the field potential duration, were compared with the analogous values obtained from manual analysis. The reliability of the correlation analysis algorithm, evaluated using various arrhythmogenic and morphology changing signals, revealed a mean sensitivity and specificity of 99.27% and 94.49% respectively, in determining true field potential complexes. The field potential duration of the averaged waveforms corresponded well to the manually analysed data, thus demonstrating the reliability of the software. The software has also the capability to create overlay plots for signals recorded under different drug concentrations in order to visualize and compare the magnitude of response on different ion channels as a result of drug treatment. Our novel field potential analysis platform will facilitate the analysis of CM MEA signals in semi-automated way and provide a reliable means of efficient and swift analysis for cardiomyocyte drug or disease model studies.

## Introduction

Cardiac safety pharmacology testing is used to identify drug-induced complications, such as prolongation of the QT interval, due to several cardiac and non-cardiac drugs. The prolongation of QT-interval has been linked with the occurrence of severe arrhythmias which have often proved to be fatal [1] [2]. As a result of cardio-toxic effects, many drugs have been withdrawn from the market or advanced stages of preclinical drug development. To avoid such undesired consequences regulatory authorities such as Food and Drug Administration (FDA) and European Medicines Agency (EMEA) require in vitro testing for all drug candidates to reveal potential risks of QT-interval prolongation before clinical experiments.

In vitro preclinical testings have shown to reduce cost, time and failed clinical trials [3] [4]. Multi-electrode arrays (MEAs) can be used to study cellular electrophysiology of cardiomyocytes (CMs) at the cell population level. The use of MEA is a well-accepted technique for recording electrical signals from excitable cells and tissues with high spatial and temporal resolution [5] [6]. The cell culture dish with MEA has surface embedded electrodes that can sense changes in the electrical activity of the cells. The signals recorded are extracellular field potentials generated by the CMs [7]. Earlier studies have shown that the extracellular field potential recordings can be used to determine characteristics of the cardiac action potential such as the field potential duration (FPD), which correlates closely with the QT-interval in the electrocardiogram (ECG) [8] [9]. As a result, the MEA platform has been used extensively in the study of human pluripotent stem cell (hPSC) derived CMs [10]–[13] and in vitro electrophysiological drug testing [14]–[17].

MEA recordings can generate large volumes of data as several electrodes from each individual recording contain useful information. Traditionally, data from MEA recordings have been analysed manually which is labour-intensive, slow and often user dependent. Manual data analysis forms a bottleneck for high-throughput screening and can sometimes be unreliable due to poor quality of the signals. Commercial software for CM MEA data analysis (Cardio2D+, Multichannel Systems Reutlingen, Germany),generic software (e.g. Spike2 – Cambridge electronic design, Labchart – AdInstruments, ClampFit – Molecular devices) that can be utilized for cardiomyocyte MEA data analysis or other assay services (QTempo, Reprocell, Japan) are currently available for cardio toxicity testing but most of them suffer certain drawbacks. For example, the Cardio2D+ analysis software allows averaging of multiple field potential complexes for data analysis but requires its own specialized recording software. Moreover, it requires data to be in its native format, thereby rendering data from other sources incompetent. MATLAB (Mathworks, Inc., Natick, Massachusetts, United States) based programs and some custom-made MATLAB toolboxes [19]–[22] have been developed for signal analysis but these programs require MATLAB to run and, with the exception of MEA-tools, they are not available in open source. MEA recordings from three-dimensional CM aggregates exhibit variation in signal amplitude and shape due to distance and orientation between the source and the electrode surface [18]. As a result, the signal-to-noise ratio (SNR), depicting the quality of the recorded signals, may vary between different cardiac cell populations. Moreover, the detection of sporadically occurring arrhythmic complexes also poses a challenge to researchers, who often have to screen large volumes of data to identify such morphology changes as a result of drug treatment.

The aim of the study was to develop a field potential analysis software platform to facilitate evaluation of drug response in CMs and compare the FPD prolongation against different drug concentrations. Here, we present our offline, semi-automatic software ‘Cardiomyocyte MEA Data Analysis (CardioMDA)’, which is designed to improve the accuracy, reliability and throughput rate for drug screening and analysis. We demonstrate the usability and capability of the software using human induced pluripotent stem cell (iPS cell) derived CMs. The software is validated by calculating the mean sensitivity and specificity of true field potential detection using several arrhythmogenic and morphology changing signals. In addition, the software based analysis outcomes are compared to manual analysis results to establish the reliability of the analysis software.

## Materials and Methods

### Human iPS cell generation, cell culture and cardiac differentiation

The study was approved by the ethical committee of Pirkanmaa Hospital District (R08070) and written informed consent was obtained from all the participants. Human iPS cells were generated as described earlier [23]. Control human iPS cells (UTA.04602.WT) [24] were cultured in knockout serum-replacement (KSR) medium using mouse embryonic fibroblasts (MEF; Millipore, Billerica, MA) as feeders. The components of KSR medium are: knockout (KO)-DMEM (Invitrogen) containing 20% KO-serum replacement (KO-SR, Invitrogen), nonessential amino acids (NEAA), Glutamax, penicillin/streptomycin, 0.1 mM 2-mercaptoethanol and 4 ng/ml fibroblast growth factor 2 (FGF2) (bFGF, R&D Systems Inc., Minneapolis, MN, USA). The medium was refreshed three times a week and the human iPS cells were passaged weekly using collagenase IV (Invitrogen). Human iPS cells were differentiated into CMs in co-culture with mouse visceral endoderm-like END-2 cells [25] (a kind gift from Christine Mummery, Hubrecht Institute, Utrecht, The Netherlands). The END-2 differentiation method has been described elsewhere [25].

### In vitro field potential recordings and data analysis

Beating aggregates of CMs were manually dissected and plated on 6-well MEAs (6-well MEA 200/30iR-Ti-tcr, Multichannel Systems, Reutlingen, Germany), which were first coated with fetal bovine serum (FBS, Invitrogen) for 30 minutes at room temperature and then with 0.1% gelatine (Sigma Aldrich) for 1 hour at room temperature. The CM aggregates were cultured in EB-medium: KO-DMEM with 20% FBS, NEAA, Glutamax and penicillin/streptomycin. The field potentials of spontaneously beating aggregates of CMs were recorded at +37°C with the MEA platform (MEA1060-Inv-BC, Multichannel Systems, Reutlingen, Germany) using 20 kHz sampling frequency and MC_Rack (Multichannel Systems, Reutlingen, Germany) software. The MEAs were covered with gas-permeable membranes (ALA MEA-SHEET, ALA Scientific) during recordings. For drug tests, the CMs were cultured in 5% FBS containing EB medium. The hERG channel blocker E-4031 (Sigma Aldrich), Sotalol (Sigma Aldrich), JNJ303 (Tocris Bioscience) and Esmolol (Brevibloc, Baxter) were diluted in culture medium for a concentration range of 100–700 nM, 1.6–19.4 µM, 1–7.5 µM and 84–168 µM respectively. Baseline conditions as well as drug effects were recorded for 2 minutes after a 2 minute drug wash-in. The FPDs and beating rates from the recordings were analysed offline by ClampFit (Molecular Devices) and by our CardioMDA software for comparison studies. Twenty five field potential complexes for each individual recording from the raw data were analysed manually and the mean FPDs were compared to the corresponding averaged signal FPDs obtained using CardioMDA.

### System requirements and data format

CardioMDA is available in the executable file format and requires about 6 Mb of free hard disk space. The program will run on any system running Matlab 7.13 (The MathWorks Inc., Natick, Massachusetts, United States) or a system installed with the Matlab Compiler Runtime version 7.16 (The MathWorks Inc., Natick, Massachusetts, United States). The current version has been tested on Matlab R2011b running on Microsoft Windows 7 (Microsoft Inc., Seattle, United States) platform and is available in both 32-BIT and 64-BIT file formats.

The signals of CMs recorded using MEA1060-Inv-BC amplifier (Multichannel Systems Reutlingen, Germany) or similar, are in the native. mcd format. The current version of the program will only support files in the ASCII format, converted using the MC_DataTool (Multichannel Systems, Reutlingen, Germany) software. Signals from multiple electrodes can be recorded on a single ASCII file with header lines containing information on the available electrodes, which is essential during analysis.

### Program description

**Figure 1:**
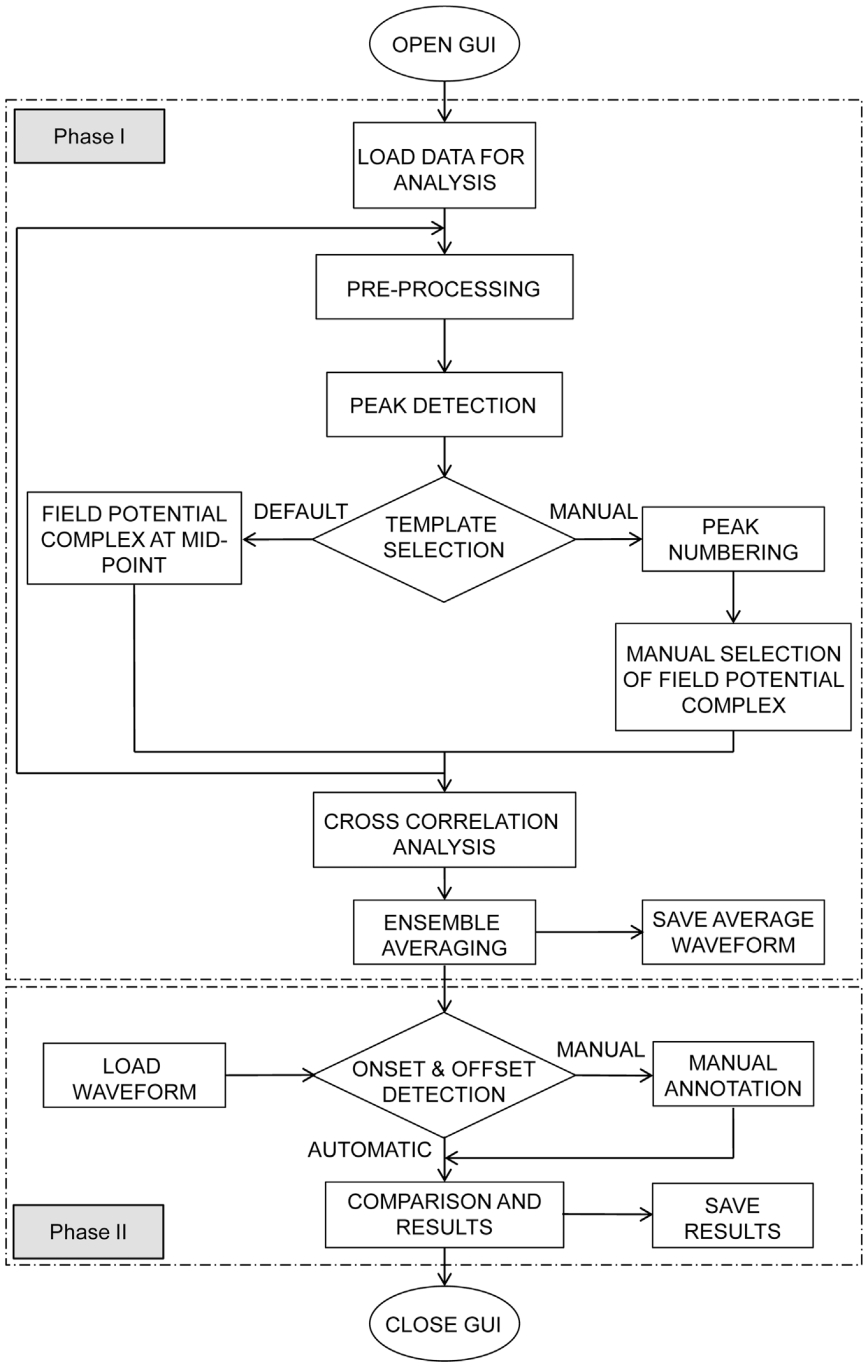
Flowchart representing the workflow of CardioMDA. On execution of the program, the graphical user interface (GUI) opens up. In phase I, the user loads the data that is to be analysed. The program automatically pre-processes to remove noise and after peak detection, the user is prompted to select a template for analysis. During correlation analysis, the program checks for field potential complexes whose correlation coefficient satisfies the user-defined correlation factor and produces an average waveform. In phase II, the program allows the user to define the onset and offset, either automatically using the built-in algorithms, or manually by the user. The process is repeated for upto ten signals and the field potential duration and other parameters are compared. The results can be saved for future reference.

The flowchart of the CardioMDA module is shown in Fig. 1. On execution of the program and opening of the graphical user interface (GUI), the first step involves importing data that needs to be examined. This can be performed at two different stages: Phase I – for correlation analysis and averaging or Phase II – for evaluation of FPDs.

In Phase I, raw signals are imported for analysis and the available electrodes are indicated on the program interface to assist the user in choosing electrodes that needs to be analysed. After pre-processing and peak detection, a template, which is used as a comparison standard all detected field potential complexes, is selected for cross correlation analysis and ensemble averaging. In phase II, signals that have already undergone Phase I processing are imported for in-depth analysis and quantization of FPD prolongation due to drug injection. Up to ten different signals can be analysed simultaneously in the phase II stage.

#### Pre-processing

Low-pass Butterworth filter of order 5 with a cut-off frequency of 200 Hz is implemented to attenuate noise that may affect signal quality. For correlation analysis, it is essential that all the field potential complexes in the signal are detected accurately. A simple window-threshold based technique has been adapted for our software.

#### Correlation analysis

Correlation analysis plays an integral role in identifying arrhythmogenic or morphology changing signals as a result of drug treatment. Correlation analysis is performed to identify field potential complexes that closely resemble the selected template [26] [27]. In our program, the template can either be chosen as default, which denotes the selection of the field potential complex positioned at the centre of the total recording duration, or manually, allowing the user to select a field potential complex that best reflects the effect of drug on the cells. The cross correlation function then measures the degree of similarity between the chosen template and all detected field potential complexes. The correlation coefficient is computed as the sum of the products of corresponding pairs of points from the two complexes within a specified time window. The formula for cross correlation is represented as:
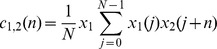
Where *N* represents the number of samples in the time window, *n* is the overlap factor and *x_1_* and *x_2_* are the signals. The factor *1/N* is used to normalize the results based on the number of sample points. The value of *c_1,2_* determines the level of similarity between the two signals. A user-defined correlation factor (CF) determines the level of similarity required to pool the data for averaging. All the field potential complexes whose correlation coefficients are equal or greater than the pre-defined CF are ensemble averaged to produce the representative field potential complex. The resulting average waveform has been shown to represent a general morphology of the field potential complexes present in the raw signal [28] [29].

#### Field potential duration onset and offset detection

The detection of the beginning and end-point of the FPD (onset and offset points, respectively) is essential for reliable data analysis. Here, automatic onset detection was formulated based on amplitude changes at every sample point in the signal with respect to subsequent amplitudes within a particular time frame. Trapezium's area method (TRA) proposed by Vázquez-Seisdedos*et*al.[30] to identify the end of T wave is adapted for offset detection and is based on calculation of successive areas of a rectangular trapezium with three fixed vertices and one moving point vertex. The moving point vertex shifts from detected valley-point after the activation peak to the last available sample point while the total trapezium area is computed and the point at which the area is maximum can be defined as the offset. From the onset and offset points, the program computes the FPD, corrected FPD (cFPD) using Bazett's equation and the area under the curve. Bazett's equation is used to normalize FPD measurements to the beating rate of contracting cardiac cells [15].

### Graphical User Interface (GUI)

**Figure 2:**
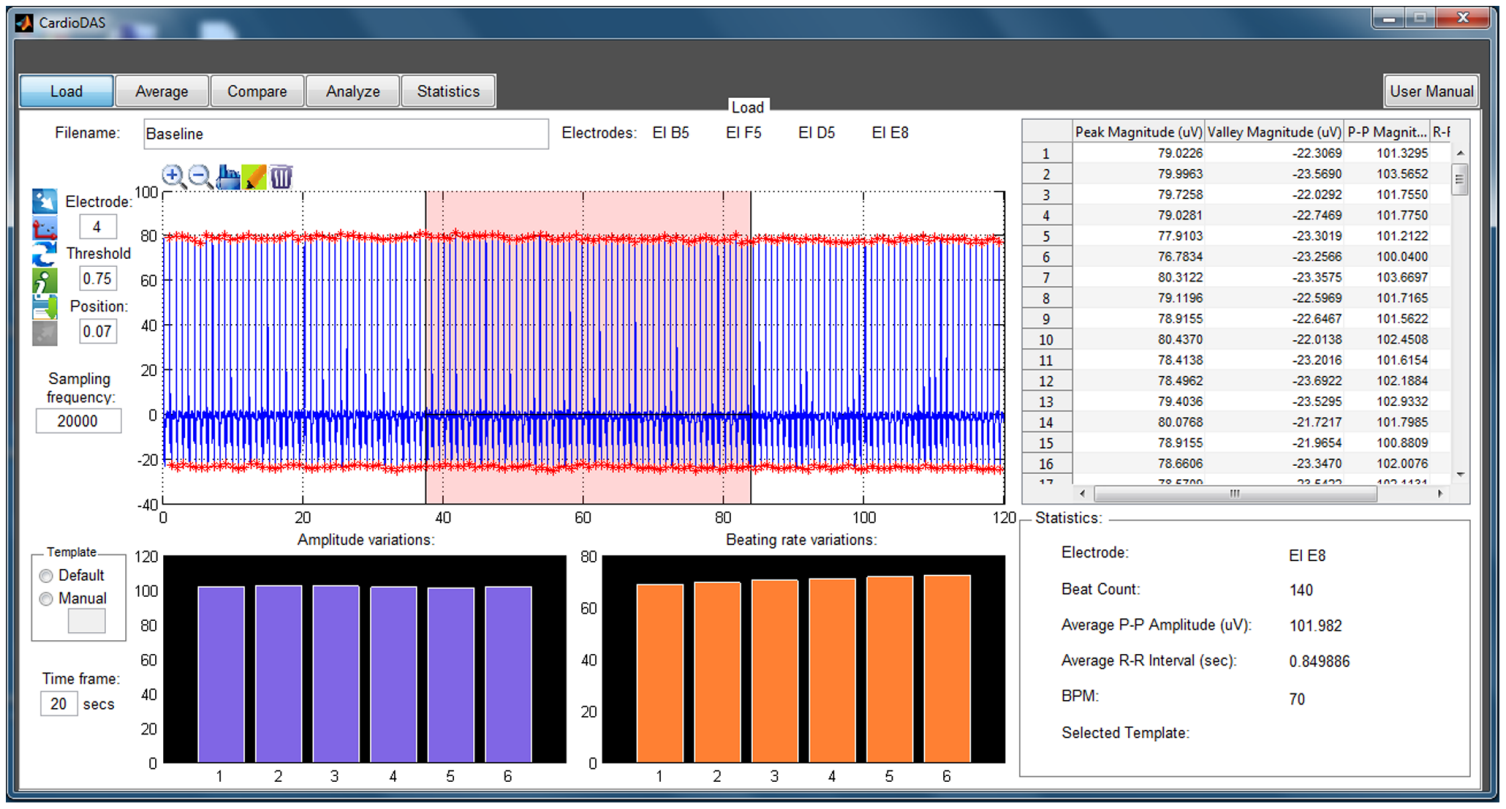
Screenshot of the graphical user interface for CardioMDA. The screenshot presents the *Load* panel of the CardioMDA software with its interactive interface options and basic signal information. Once the template has been selected, the user then moves to the *Average* panel to set and execute the correlation analysis. *Compare*, *Analyze* and *Statistics* panel are used to measure the field potential duration and compare it with other drug concentrations.

The interface is divided into five panels: The *Load* panel for data and signal information, *Average* panel for the correlation analysis, *Analyze* panel for onset and offset detection, *Compare* and *Statistics* panels to study the properties and differences between drug concentrations. Each panel has its own set of icon buttons to perform different functions. The tool tip strings describe the basic function of each user interface button, thereby assisting new users for ease of operation. All analysis results and overlay plots can be saved for future reference. Fig. 2 shows the screenshot of CardioMDA GUI with the load panel on display.

### Statistical analysis

Sensitivity and specificity analysis are used to evaluate the performance of the correlation analysis technique used in this study. In order to define these measures, it is essential to have a fundamental understanding of the following terms: *TP* – number of true positives (field potential complex resembles the selected template and the correlation coefficient is equal or greater than CF); *FP* – number of false positives (field potential complex does not resemble the template selected but the correlation coefficient is greater than or equal to CF); *TN* – number of true negatives (field potential complex does not resemble the template and the correlation coefficient is lesser than CF); and *FN* – number of false negatives (field potential complex resembles the template but the correlation coefficient is lesser than CF). Sensitivity is the proportion of field potential complexes which satisfy the CF and are correctly classified as those which adequately resemble the selected template.
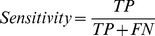



Specificity is the proportion of complexes which do not satisfy the CF criterion and are correctly classified as dissimilar to the template.
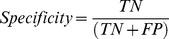



The positive predictive value (PPV) signifies the proportion of field potential complexes with a positive CF which actually resemble the selected template.
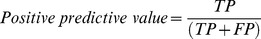



## Results and Discussion

Cardiac drug safety screening is of big concern in the pharmaceutical industry. The withdrawal of several unsafe drugs from the market accentuates the need for systemic screening during the drug development phase. Analysing methods include organ models such as Langendorff heart and cell models such as conventional electrophysiology with patch clamp systems on CM and heterologous expression systems [8]. Traditionally, the patch clamp technique has been regarded as the golden standard to determine electrical properties of the cells but it needs highly skilled operator and is time intensive, therefore making it unsuitable for high-throughput screening [4]. Moreover, currently available methods are not fully adequate to reveal adverse repolarisation effects of potential new drug candidates [31] [32]. The MEA platform together with hPSC-derived CMs may serve as a complement to current methods for screening new drugs. The recent hardware development in the MEA-technology has led to the capability of acquiring large volumes of data. For example, hardware from Multichannel Systems (MCS, Germany) allows recording of electrical activity from up to 252 electrodes (USB-MEA256) and up to nine different cell populations (256–9 well MEA) at once. Although the actual MEA recordings are relatively simple and time-efficient, the development of software for data analysis has fallen behind. The manual data analysis is demanding and has been a bottleneck for high-throughput screenings. To perform these screening tests in a time efficient and systemic manner, we formulated the CardioMDA that is intended to be useful for researchers working with CMs. The software is interactive and requires no specific programming knowledge as all the functions are available on the intuitive graphical user interface.

### Correlation analysis and ensemble averaging

**Figure 3:**
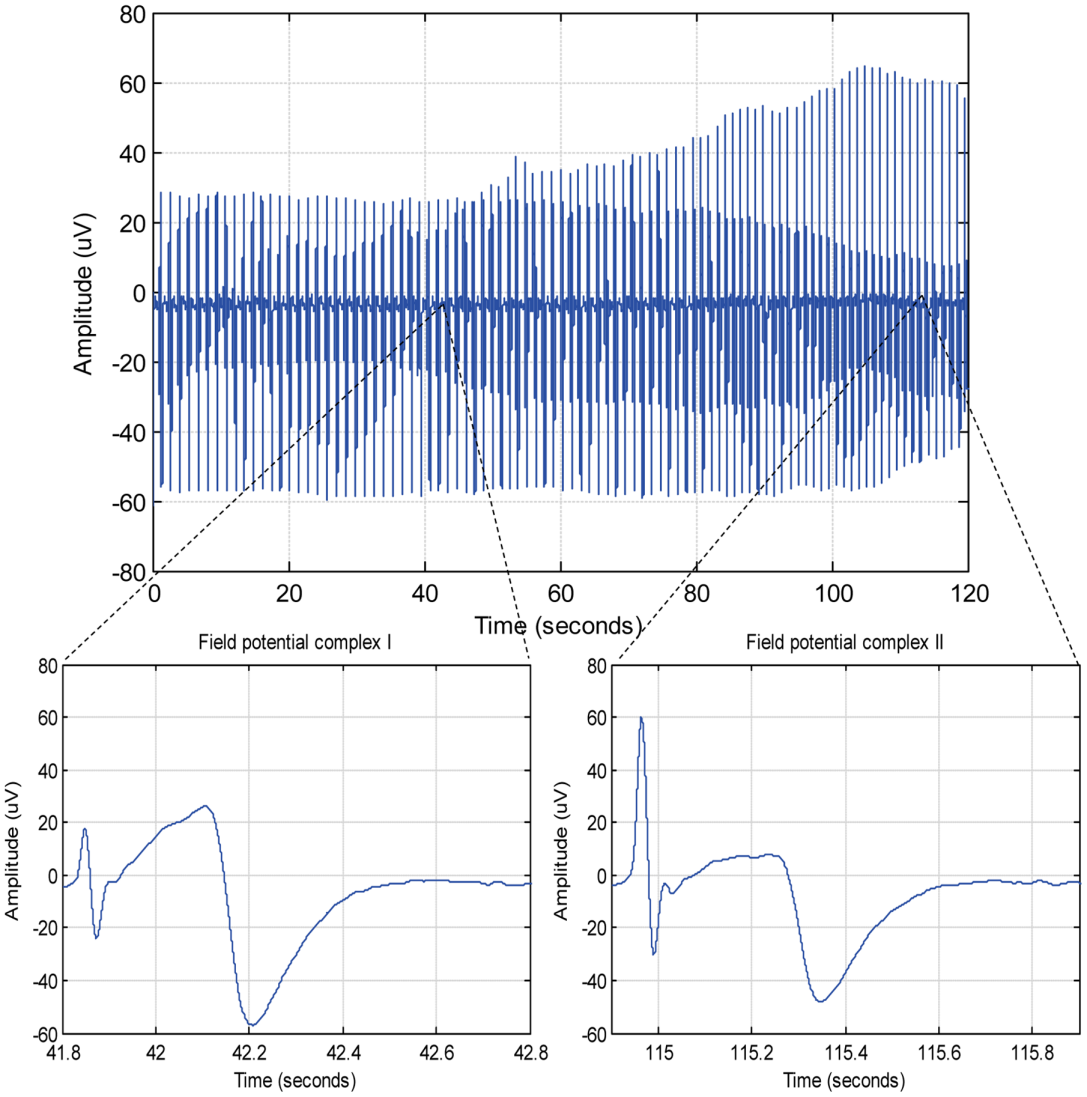
Unstable signal morphology as a result of drug treatment. Signal showing changing morphology as a result of drug treatment. Field potential complex I and field potential complex II have different signal properties in terms of amplitude and duration, thereby accentuating the need for correlation analysis.

Although several studies have successfully adapted the correlation technique for ECG beat detection and location of fiducial points (landmarks of ECG such as onset of P wave) [27] [33] [34], the correlation algorithm has not been tested on electrical activity recorded from CMs plated on MEA. The significance of using this method lies in its ability to accurately and consistently determine true field potential complexes in morphology changing or arrhythmogenic signals. Likewise, by choosing an arrhythmogenic field potential complex as template, the incidence of arrhythmogenic behaviour in the recording can be determined. This greatly simplifies the task of manually screening the datasets to identify arrhythmogenic behaviour. Fig. 3 shows an example of how the drug affects the morphology of the signal and the importance of choosing an appropriate template for the analysis.

**Figure 4:**
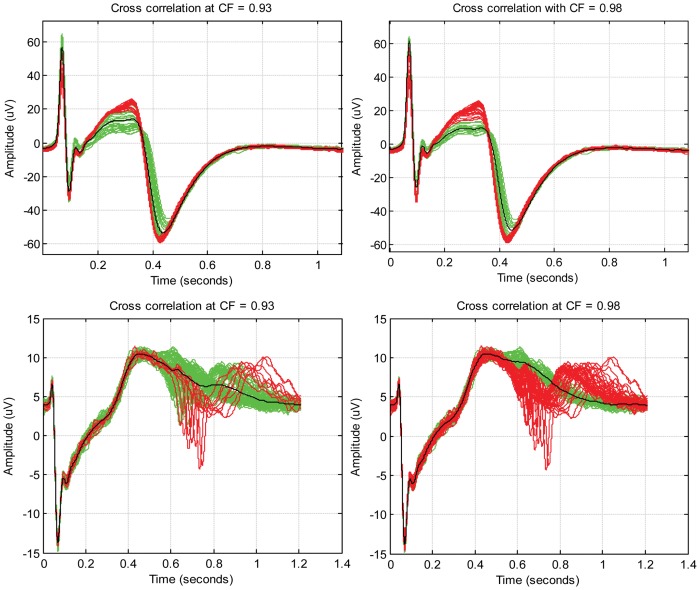
Comparison of correlation analysis results with different values of correlation factor. An illustration of the correlation analysis procedure for morphology changing (top row) and arrhythmogenic signals showing premature activation (bottom row) for different correlation factors is shown. The field potential complexes in green have a correlation coefficient greater than correlation factor and are identified as true field potential complexes (accepted for averaging) whereas those in red are identified as true negatives (rejected for averaging). The black line presents the morphology of averaged signal. In both cases, it can be seen that for lower value of correlation factor (0.93), a number of false positives occur, causing a significant change in the morphology of the averaged waveform. From the bottom row, it can be seen that a higher correlation factor (0.98) is more accurate in identifying the true field potential complexes.

To evaluate the results of correlation analysis, stable, morphology changing and arrhythmogenic signals were examined and were subject to analysis with different values of correlation factors (CFs). Arrhythmogenic signals showing premature activation were obtained by applying a hERG potassium channel blocking drug E-4031 to the CM aggregates. Morphology changing signals (see example in Fig. 3) were obtained when E-4031 challenged CM aggregates were treated with β-blocker Esmolol. The same template was chosen each time for different signal set (normal, morphology changing and arrhythmogenic signals) and analysed using different CF values. Fig. 4 shows how the identification of true field potential complexes is affected by CF. Sensitivity and specificity of determining true field potential complexes were calculated for these datasets, as shown in Table 1, to ensure the results are consistent. It can be observed that with increasing CF, there is a significant increase in positive predicted value (PPV) and specificity, irrespective of the type of signal being analysed.

**Table 1:** Cross correlation results for normal, arrhythmogenic and morphology changing signals with different values of correlation factors.

MEA recording from hiPSC-CMs	CF	TP	FP	TN	FN	PPV	Sensitivity	Specificity
**Normal signal**								
Baseline	0.88	136	2	0	0	98.55%	100%	–
	0.93	136	2	0	0	98.55%	100%	–
	0.98	136	0	2	0	100%	100%	100%
**Morphology changing signal**								
E-4031 400 nM + Esmolol – 84 µM	0.88	38	20	0	0	65.52%	100%	–
	0.93	38	6	14	0	86.36%	100%	70%
	0.98	38	2	18	0	95%	100%	90%
**Arrhythmogenic signal**								
E-4031 – 700 nM	0.88	59	27	0	0	68.61%	100%	–
	0.93	59	7	20	0	89.39%	100%	74.07%
	0.98	59	3	24	0	95.16%	100%	88.89%

CF-Correlation factor, TP-True positive, FP-False positive, TN-True negative, FN-False negative, PPV-Positive predictive value.

From the table, it can be seen that PPV and specificity show significant improvement with an increase in CF. Experimental results have shown that CF between the ranges of 0.96–0.98 produce consistent results.

To ensure the algorithm produces consistent results, analysis was performed on multiple datasets. Table 2 presents the outcome from this study. The mean sensitivity and specificity for accurately detecting true field potential complexes with CF set at 0.98 were 99.27% and 94.49%, respectively. The algorithm also exhibited a mean PPV of 96.35%, which indicates that the detection algorithm is accurate. However, extremely high CF may render a very rigid selection criterion, greatly reducing the number of ensembles available for averaging. Therefore, the CF should be chosen prudently such that a right balance is achieved between statistical performances and averaging. From experiments, it was determined that CF values in the range of 0.96–0.98 were optimal for correlation analysis. However, the user needs to consider the degree of similarity that needs to be attained between the selected template and field potential complexes to satisfy the selection criterion. In some studies, the signal shape may prove to be trivial and therefore a less stringent CF will suffice the study protocol.

**Table 2:** Statistical measure of performance in accurately detecting true field potential complexes in arrhythmogenic and morphology changing signals using cross correlation with CF = 0.98.

MEA recording from hiPSC-CMs	TP	FP	TN	FN	PPV	Sensitivity	Specificity
**Arrhythmogenic signals**							
E-4031 – 10 nM	141	0	9	0	100%	100%	100%
E-4031 – 100 nM	281	3	24	2	98.94%	99.34%	88.89%
E-4031 – 700 nM	59	3	24	0	95.16%	100%	88.89%
E-4031 – 700 nM	22	2	51	0	91.67%	100%	96.23%
**Morphology changing signals**							
E-4031 400 nM + Esmolol 84 µM	38	2	18	0	95%	100%	90%
E-4031 – 700 nM + Esmolol 84 µM	37	2	71	2	94.87%	94.87%	97.26%
E-4031 – 700 nM + Esmolol 168 µM	37	1	167	0	97.36%	100%	99.41%
E-4031 – 1000 nM+ Esmolol 168 uM	44	1	20	0	97.78%	100%	95.24%
**Average:**					**96.35%**	**99.27%**	**94.49%**

CF-Correlation factor, TP-True positive, FP-False positive, TN-True negative, FN-False negative, PPV-Positive predictive value.

Sensitivity, specificity and PPV show consistent results for different datasets of arrhythmogenic and morphology changing signals. Results indicate that the correlation analysis is highly sensitive and specific in detecting field potential complexes. A mean PPV of 96.35% indicates that true field potential complexes are detected with a high degree of accuracy, irrespective of the type of signal being analysed.

Cross correlation has been used extensively for beat detection and averaging ECG waveforms to remove artefacts [35]–[36]. Variations from the cross correlation algorithms in ECG have also been successful and computationally efficient [37]. However, to our knowledge these tools have not been used in MEA analysis. The correlation interval is an important parameter to consider while making correlation analysis. If the interval is too large, the components of the adjacent field potential complex maybe considered for correlation whereas, on the other hand, if the interval is too small, the desired portions of the signal may not be considered [27]. In order to ensure that such discrepancies are avoided, we chose the smallest beat-beat duration as the correlation interval.

**Figure 5:**
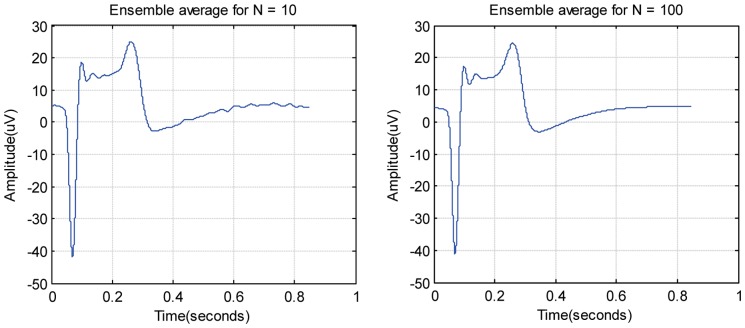
Illustration of smoothing effect after ensemble averaging. *N* denotes the number of ensembles used for averaging. The resulting waveform from ensemble averaging is smoother when the number of ensembles being averaged is higher.

Ensemble averaging has been shown to be effective for noise attenuation [38]. Higher number of ensembles produces a better SNR which conceives a smooth average of the field potential complexes. For ensemble averaging, precise synchronization of field potential complexes is vital. The averaging accuracy depends on the accurate definition of fiducial points in the field potential complexes and the constant time interval between the point and signal being analyzed. In our study, the normal field potential complexes were aligned with the window centred on the detected QRS-like complex as the variability between beats was found to be minimum. The smallest beat-to-beat interval was chosen as the window length for the alignment. The number of ensembles recorded during a period of 2 minutes from each cardiac aggregate varied across different recordings. As illustrated in Fig. 5, it was observed that the signal was smoother as the number of ensembles increased. However, inaccurate alignment of the ensembles could cause a low pass filtering effect [39] which might suppress the high frequency components during averaging. Laciar et al. [40] have shown that an alignment error of 1 millisecond (ms) corresponds to a low pass filter of cut-off frequency 133 Hz for ECG measurements. In order to avoid filtering effects of ensemble averaging, it is ideal that the alignment errors are less than 0.5 ms [40].

### Field potential duration measurements

Although several approaches have claimed to be successful in accurately determining the onset and offset of ECG [41]–[46], they have limited utility in the MEA field potential analysis as the MEA depolarisation and repolarisation phases may exhibit much more diverse waveforms with narrow amplitude gradients and several diverse morphological features: positive, negative, only upwards, only downwards or biphasic waveforms. One source of variability could be the three-dimensional CM aggregates used in MEA analysis. These aggregates exhibit more variation in signal shape between the recordings and between the different electrodes due to their three-dimensional structure and spatial variability with respect to the MEA electrodes. This constitutes a major challenge in developing robust automated algorithms for the analysis of CM MEA signals. Due to such inherent variability in geometry, the onset and offset determined using our indigenously developed automatic detection algorithms have been ambivalent. Another possible cause for ambiguity could be due to differences in perception. Different studies have shown that researchers have chosen these fiducial points based on individual reasoning and observation. For example, some studies have considered repolarisation wave peak as the offset whereas several other researchers have chosen the absolute end of the repolarisation phase to determine the FPD [17] [47] [48]. In order to overcome these issues, the CardioMDA facilitates the use of operator's discretion to decide between automatic/manual determinations of the onset and offset.

We investigated the reliability and efficiency of the CardioMDA in determining the FPD of CMs by applying known hERG potassium channel blocker E-4031 [41] to the human iPSC derived CMs and comparing the results to the manual analysis. As a result of hERG channel block, the FPD is prolonged due to increasing concentration of the drug [15] [16]. The signals recorded after every drug challenge were averaged and manually annotated for onset and offset using our software. Concurrently, the raw signal data for each drug concentration was analysed manually and compared with the results obtained from the averaged signal. Table 3 shows the comparison results between the raw signal data and averaged data. The mean and standard deviation (S.D.) of FPD from the raw signal data rises due to the increasing E-4031 concentration. The FPD measurements obtained from the averaged signal corresponded well with the raw signal measurements, indicating that the results from the CardioMDA are consistent.

**Table 3:** Field potential duration prolongation with increased hERG channel blocker E-4031 drug concentrations.

	Manual analysis of raw data		Analysis using CardioMDA	
	Mean ± S.D.		Mean	
E-4031 drug concentration	FPD (ms)	BR (bpm)	FPD (ms)	BR (bpm)
Baseline	300.34±8.81	128±2.4	306	127
E-4031–100 nM	306.05±9.84	135±2.5	313.5	134
E-4031–300 nM	329.57±10.30	130±2.0	331.2	129
E-4031–500 nM	360.50±10.7	122±1.2	364.1	122
E-4031–700 nM	410.93±14.0	113±0.86	423.9	112

Abbreviations: FPD- field potential duration, BR-beating rate.

Mean and S.D. of field potential durations from manually analysed field potential complexes (n = 25) were compared to those of corresponding averaged signals from CardioMDA in a drug dataset depicting the effect of E-4031 on human iPSC-derived CMs. The field potential duration rises as a result of increasing drug concentration. From the results obtained using our software, it can be seen that the changes in field potential duration correspond well to values obtained from manual analysis.

**Figure 6:**
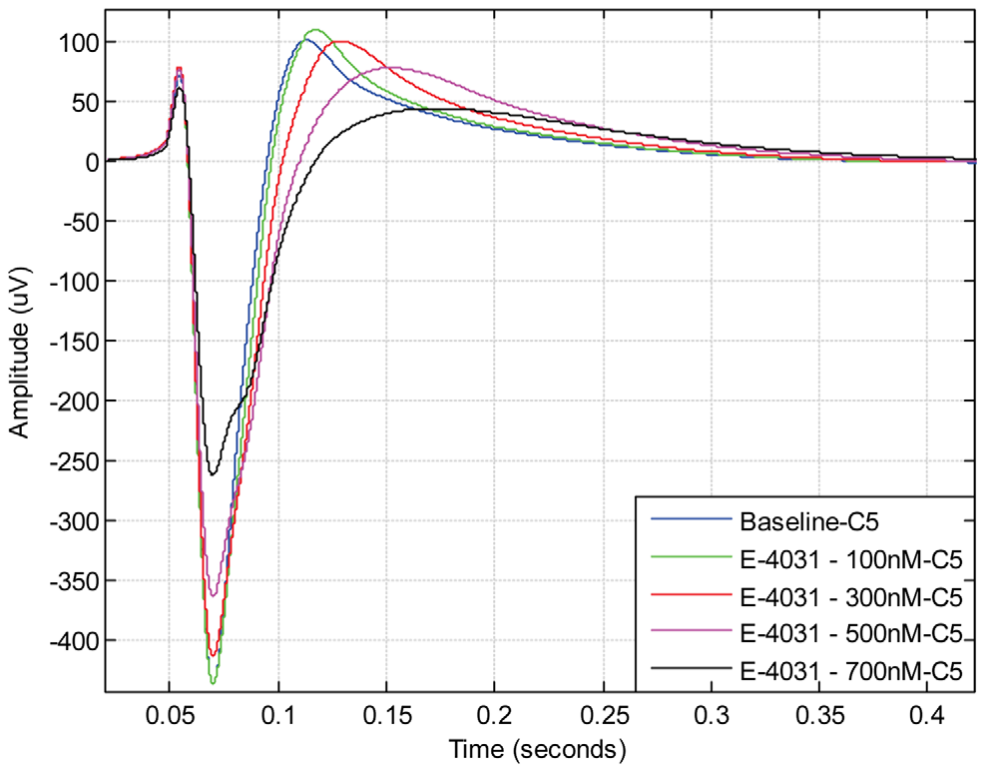
Overlay plot showing the effect of hERG potassium channel blocker E-4031 on field potential duration. As a result of E-4031 treatment, the increase in field potential duration is dependent on the concentration of the drug. The effect of the drug can most prominently be seen at the repolarisation wave peak as a change of signal amplitude and the prolongation of the repolarisation wave peak compared to baseline signal. The effect of the drug can also be seen as a prolongation of the offset point at the end of the field potential cycle.

Despite having statistical information of the FPD prolongation, the differences in FPD do not reveal either the dynamics of different ion channels or the magnitude of response as a result of drug treatment. To be able to better understand these changes in the ion channels, we have incorporated an overlay chart, which allows up to 10 different signals recorded under different drug concentrations, to be plotted for visual comparison. Fig. 6 is an example of the overlay chart, which depicts the effect of the hERG blocker E-4031 used in our experiment. From Fig. 6, it can be observed that the FPD and repolarisation wave peak is prolonged, and the amplitude of the initial sodium spike and repolarisation wave (which corresponds to the ECG T-wave) decreases as a result of increasing E-4031 concentration.

**Figure 7:**
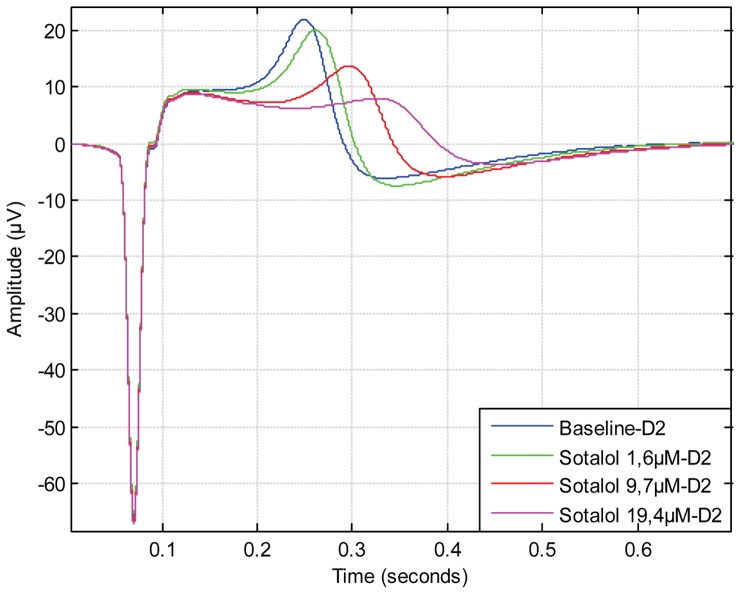
Overlay plot showing the effect of sotalol on field potential duration and signal shape. As a result of drug treatment, the repolarisation wave peak, which corresponds ECG T-wave, is prolonged due to increasing concentration of Sotalol. However, the offset point of field potential duration is similar between different Sotalol concentrations.

**Figure 8:**
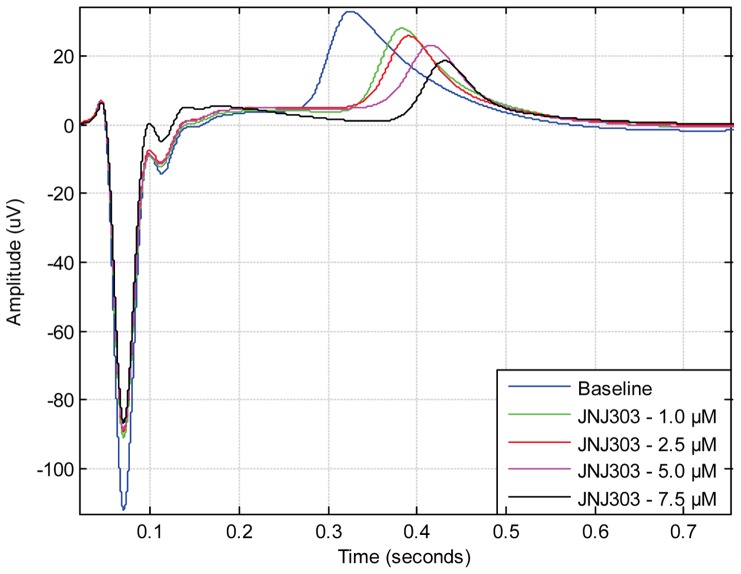
Overlay plot showing effect of IK_s_-blocker JNJ303 on field potential duration and signal shape. Similar to Sotalol, JNJ303 prolongs the repolarisation wave peak but the field potential duration offset point is similar between different JNJ303 concentrations.

The offset point of the field potential cycle is most substantial when assessing the effect of cardiac ion channel blocking drugs as the FPD correlates with the QT-interval in ECG [8] [9]. Although most drugs have been shown to prolong the FPD [15] [16], some drugs do not seem to have effect on the offset point of the field potential cycle. Rather, the effect is concentrated on the initial repolarisation wave phase as seen in the case of anti-arrhythmic drug Sotalol (Fig. 7) and potassium channel IK_s_ blocking drug JNJ303 (Fig. 8). When analysing these types of signals manually, it is hard to distinguish repolarisation wave prolongation for different drug concentrations. With the CardioMDA overlay feature, these acute changes in morphology can be observed. The prolongation of the repolarisation wave due to increasing drug concentration could be used to determine the relative QT-interval prolonging effect of drugs on CMs.

## Conclusion

CardioMDA has been shown to be suitable for analysing MEA signals from human iPS cell derived CMs, which can be used in the pre-clinical context for assessing the safety of new drugs or screen candidate drugs in patient-specific cardiac disease modelling. Traditionally, the FPD is determined manually by examining several field potential complexes from the raw data or averaging determined number of field potentials [38]. Our solution is to use cross correlation method to identify the representative field potential complexes based on a pre-selected templates. These methods enable automatic determination of single or multiple field potential complexes to represent the data for further detailed analysis and visualization. The accurate determination of onset and offset for calculating FPD from raw signals is often tedious due to noise. Moreover, manual data analysis has proven to be time-consuming, work-demanding and the results are usually user biased. Based on the template method, these discrepancies can be overcome and it provides a means for reliable FPD analysis. Apart from eliminating ambiguity in the results obtained manually, there are several benefits with the use of CardioMDA. The software is capable of screening large datasets when compared to manual analysis and ensures increased work and time efficacy as only one averaged signal representing each cardiac cell population needs to be analysed. At present, we are not aware of any similar software that is available in open source. As there is increased use of hPSC-derived CMs for drug screening with MEA [15] [16] [49] [50], there is a significant demand for faster and consistent analysis methods. We believe our software will provide a reliable means of efficient and swift analysis for future studies.

### Availability

The CardioMDA has been designed to facilitate the data analysis of MEA recordings obtained from CM cell aggregates and is available for registered users. Website for downloading the program: www.biomeditech.fi/CardioMDA.
